# Personality Change Profiles and Changes in Cognition Among Middle-Aged and Older Adults

**DOI:** 10.1093/geroni/igab046.3240

**Published:** 2021-12-17

**Authors:** Mirjam Stieger, Yujun Liu, Eileen Graham, Jenna DeFrancisco, Margie Lachman

**Affiliations:** 1 Brandeis University, Waltham, Massachusetts, United States; 2 Northern Illinois University, Naperville, Illinois, United States; 3 Northwestern University, Chicago, Illinois, United States; 4 Brandeis University, Brandeis University, Massachusetts, United States

## Abstract

Previous research on the relationship between personality traits and cognitive abilities has primarily focused on cross-sectional studies or on specific personality traits in relation to selected cognitive dimensions. The present study extends existing research by exploring associations among 20-year personality change profiles and 10-year cognitive change in middle-aged and older adults. The present study included 2,652 participants of the Midlife in the United States study (MIDUS) ranging in age between 20 - 74 years (M = 46.61, SD = 11.26) at the first of the three measurement occasions. Latent Profile Analysis (LPA) was used to capture profiles of change across the Big Five personality traits of extraversion, conscientiousness, agreeableness, openness, and emotional stability combined. Results of the LPA identified three personality change subgroups: Decreasers, Maintainers, and Increasers. Across the 20 years, the Decreasers showed greater decreases on the Big Five personality traits, the Maintainers remained mostly stable, and the Increasers showed greater personality trait increases. Also, the Maintainers and Decreasers were significantly older than the Increasers. Longitudinal multilevel models were used to examine the relationship between these three personality change profiles and cognitive change. Age, sex, education, physical activity, functional health, and self-rated health were added as covariates. Results show that cognitive decline was greater for the Decreasers and less for the Increasers compared to the other personality change profiles. The results have implications for developing interventions to target personality trait change in middle and later adulthood as a potential means for reducing declines in cognitive functioning.

